# Oncogenic potential of BEX4 is conferred by Polo-like kinase 1-mediated phosphorylation

**DOI:** 10.1038/s12276-018-0168-0

**Published:** 2018-10-22

**Authors:** Jin-Kwan Lee, Geun-Hyoung Ha, Hyun-Soo Kim, Chang-Woo Lee

**Affiliations:** 1 Department of Health Sciences and Technology, SAIHST, Sungkyunkwan University, Seoul, 06351 Republic of Korea; 2 Department of Molecular Cell Biology, Samsung Medical Center, Sungkyunkwan University School of Medicine, Suwon, 16419 Republic of Korea

## Abstract

The brain-expressed X-linked 4 (BEX4) gene has been recently identified as a mediator of microtubule hyperacetylation through sirtuin 2 inhibition and is highly overexpressed in human cancers. However, the gain-of-function molecular mechanism of the *BEX4* gene in human cancers still needs to be elucidated. This study shows that BEX4 colocalizes and interacts with Polo-like kinase 1 (PLK1) at centrosomes, spindle poles, and midbodies, particularly during mitosis. Interestingly, PLK1-mediated phosphorylation upregulates the stability of BEX4 protein, and the PLK1–BEX4 interaction allows abnormal mitotic cells to adapt to aneuploidy rather than undergo apoptotic cell death. In summary, these results suggest that the oncogenicity of BEX4 is conferred by PLK1-mediated phosphorylation, and thus, the BEX4–PLK1 interaction is a novel oncogenic signal that enables the acquisition of chromosomal aneuploidy.

## Cancer: an enabler for defective division

Tumors exploit a signaling mechanism that allows them to survive disruptions in cell division that would normally prove lethal. When normal cell division goes awry, it produces cells containing abnormal numbers of chromosomes, which exhibit numerous developmental defects. Researchers led by Geun-Hyoung Ha and Chang-Woo Lee, Sungkyunkwan University School of Medicine, South Korea, have learned how a safeguard that prevents this breaks down in cancer cells. They found that cancer cells produce elevated levels of a protein called BEX4 due to a chemical modification that renders this protein more stable. Cells that experience disrupted chromosomal sorting during cell division normally self-destruct through a process called apoptosis. Excessive BEX4 prevents apoptosis, however, allowing such damaged cells to proliferate. This fuels cancer growth but also creates opportunities for treatment with drugs that inhibit BEX4 stabilization.

## Introduction

Errors in chromosome segregation are a major cause of aneuploidy, a state where the number of chromosomes in a cell or organism deviates from multiples of the haploid genome. Aneuploidy arising during meiosis through chromosome mis-segregation is a major cause of infertility and inherited birth defects^1^. Moreover, aneuploidy during chromosome segregation can be caused by improper attachment of a chromosome to a spindle microtubule^2,3^ or weakening of the mitotic checkpoint, which delays the onset of anaphase^4,5^. The mechanism of chromosome segregation is highly complex and is mediated by microtubules. Duplicated centrosomes generate two asters of highly dynamic microtubules^6^. In addition, non-centrosomal pathways are an essential source of microtubules and are required for spindle organization and function^7^. Furthermore, finely tuned chromosome segregation depends on the coordinated changes in the assembly and disassembly of microtubules^8^.

The mitotic checkpoint promotes chromosome segregation fidelity by delaying the mitotic progression until all chromosomes are properly attached to the mitotic spindle^9^. However, some cells eventually exit mitosis after sustained mitotic arrest without mitotic checkpoint silencing, which results in multiploid progeny cells that subsequently undergo apoptosis^10^. This suggests that apoptosis plays an important role in preventing chromosomal aneuploidy from evolving into neoplastic aneuploidy. Since aneuploidy provides a growth advantage, aneuploid transformation requires disabling of the subsequent apoptosis process^4,11^. However, the mechanism that sets the apoptotic threshold whereby the fates of aneuploid cells are determined in the context of tumorigenesis remains obscure.

Our previous study showed that brain-expressed X-linked 4 (BEX4) localizes at microtubules, spindle poles, and midbodies and interacts with α-tubulin throughout mitosis^12^. The overexpression of BEX4 leads to α-tubulin hyperacetylation through the inhibition of sirtuin 2 (SIRT2) deacetylase^12^. Furthermore, we found that BEX4 expression confers resistance of apoptotic cell death but leads to the acquisition of aneuploidy, whereas increasing the proliferating potential and the growth of tumors, indicating that BEX4 acts as a novel oncogene by deregulating microtubule dynamics and chromosome integrity^12^. Moreover, BEX4 expression is highly elevated in human lung cancer cells and tissues^12,13^, and it determines whether cells undergo apoptosis or adapt to aneuploidy induced by microtubule inhibitor treatment^13^. BEX4 expression also provides resistance to microtubule inhibitor treatment by prolonged mitotic arrest and contributes to the hyper-active mammalian target of rapamycin (mTOR)-induced lung carcinogenesis^12,13^. In addition, the phenotypic heterogeneity arising from a diverse population of aneuploid cells in human tumors contributes directly to drug resistance^1^. However, the molecular mechanism of the gain-of-function of the *BEX4* gene in human cancers remains unknown.

Polo-like kinase 1 (PLK1) is a serine/threonine kinase known to have essential functions in the activation of the CDK1–cyclin B complex during the G2-to-M-phase transition, centrosome separation and maturation, spindle assembly/formation, chromosome segregation, and cytokinesis^14^. The striking feature of PLK1 is its localization to numerous subcellular structures during the process of mitosis: association with the centrosome during prophase, enrichment at kinetochores in prometaphase and metaphase, recruitment to the central spindle in anaphase, and then accumulation in the midbody during telophase^14^. PLK1 overexpression has been observed in a wide range of tumor types and is often associated with a poor prognosis including lung cancer^15^. Furthermore, *PLK1* mutations play a part in tumorigenesis^16^. A growing body of evidence indicates that the inhibition of PLK1 function leads to the prolonged mitotic arrest and subsequent apoptotic cell death^17^. Thus, PLK1 is a potential anticancer therapeutic target, and aberrant expression of PLK1 appears to be a considerable causative factor for human diseases such as cancer. This study reports that PLK1 functionally cross-talks with BEX4 in regulating microtubule dynamics and tumorigenesis.

## Materials and methods

### Cell line culture

293T and HeLa cells were cultured in Dulbecco’s modified Eagle’s medium (DMEM; WelGENE, Daegu, Korea) containing 10% fetal bovine serum (FBS; HyClone, South Logan, Utah, USA). Eleven lung cancer cell lines (WI-26, H1299, Calu-3, HCC1171, HCC1833, HCC2108, SK-LU-1, A549, HCC95, SK-MES-1, and SW900) were cultured in RPMI-1640 (DMEM; WelGENE) containing 10% FBS. To generate HeLa cells, inducible expression of green fluorescent protein (GFP) or GFP-BEX4 was performed as previously described^12^.

### Plasmid construction and transfection

Full-length human *BEX4* was generated by PCR. Full-length human *BEX4* was also subcloned into pGEX-KG (GST-BEX4) and pTAP (TAP-BEX4) for the GST pull-down assay and tandem affinity purification (TAP), respectively. Fragments encoding *BEX4* were subcloned into pEGFP-C1 (Clontech, Mountain View, CA, USA) to generate a GFP-fused BEX4 expression vector (pEGFP-BEX4). BEX4 mutant alleles were generated by site-directed mutagenesis. Comlementary DNAs of the BEX4 wild-type (WT) and five mutant versions, in which a single serine or single threonine residue was exchanged for alanine (S3A, T29A, S35A, T94A, and T107A), were subcloned into pEGFP-C1 and pGEX-KG. The pCMV-HA-PLK1 plasmid has been published previously^18^. To specifically knock down *BEX4*, short hairpin (sh)RNAs were cloned into pSuper puro (Oligoengine, Seattle, WA, USA) containing the H1 promoter according to the manufacturer’s instructions. Oligonucleotides encoding a shRNA against *BEX4* (5′-GGCCATACCTAATAGGCATAT-3′), human *CDK1* (5′- GGGGATTCAGAAATTGATC-3′), human *PLK1* (5′-GGTCCATTGGGTGTATTCATGT-3′) or the luciferase control (5′-CATACGCGGAATACTTCGA-3′) were synthesized, and the efficiencies of each shRNA were determined by immunoblotting^12,18,19^. For transient transfection, the cells were electroporated using a microporator (Digital Biotechnology, Seoul, Korea).

### Antibodies and reagents

Mouse polyclonal antibodies against human BEX4 protein were generated in C57BL/6 mice. Briefly, purified GST-BEX4 protein was injected four times intraperitoneally. Rabbit polyclonal antibodies against C-terminal polypeptides 89–106 of human BEX4 protein were commercially generated (Youngin Frontier, Seoul, Korea). The other antibodies used in this study were as follows: anti-GFP, anti-PLK1, anti-CDK1, anti-aurora A, anti-cIAP-1, anti-cdc20 (Santa Cruz Biotechnology, Dallas, TX, USA), anti-actin (Sigma-Aldrich, St. Louis, MO, USA), anti-poly(ADP-ribose) polymerase-1 (PARP), anti-cleaved-caspase 9, cleaved-caspase 7, anti-active-caspase 3, anti-phospho-threonine (p-Thr) (Cell Signaling Technology, Danvers, MA, USA), anti-aurora B (BD Biosciences PharMingen, San Diego, CA, USA), anti-securin (PTTG1; Zymed, San Francisco, CA, USA), anti-Myc (Bethyl Laboratories, Montgomery, TX, USA), and Alexa Fluor (Invitrogen, Leek, The Netherlands). The following reagents were used: MG132, cycloheximide (CHX), dimethyl sulfoxide (DMSO) (AG Scientific, San Diego, CA, USA), nocodazole (Sigma-Aldrich), and PLK1 kinase inhibitor BI2536 (Axon Medchem, Groningen, Netherlands).

### Synchronization and cell cycle analysis

To generate populations of cells with a prolonged mitotic defect, the HeLa cells were treated with different concentrations of nocodazole (200 or 300 ng/ml) and harvested at the indicated times. For the synchronization experiments, the HeLa cells were arrested at mitosis with nocodazole (200 ng/ml) for 16 h, and for flow-cytometric analyses, the cells were harvested at the indicated times post-treatment and then fixed and stained with propidium iodide. The DNA contents of 10,000 cells per sample were analyzed using a FACSCanto II cytometer (BD Biosciences).

### Recombinant adenovirus preparation and infection

Adenovirus-expressing GFP or GFP-BEX4 was generated using the pAdEasy adenoviral vector system (Stratagene, La Jolla, CA) based on the manufacturer’s instructions.

### Glutathione-*S*-transferase (GST) pull-down and in vitro binding assays

The PCR fragment encoding the *BEX4* or *PLK1* gene was subcloned into the pGEX-KG vector to generate a GST-tagged BEX4 or PLK1 construct. GST-BEX4 or -PLK1 protein was then generated in *E. coli* BL21 cells. The pull-down and in vitro binding assay method was as previously described^20^.

### Immunoblotting analysis and immunofluorescence

Phos-tag-containing polyacrylamide gels were made with Phos-tag acrylamide (20 μM; Wako, Osaka, Japan) including 10 μM MnCl_2_ with the separation gel and analyses were performed as described previously^21^. Immunofluorescence studies and other immunoblot analyses were performed as previously described^20^.

### In vitro kinase assays

Human PLK1 holoenzyme was purified from Baculovirus-infected (His-tagged PLK1) Sf9 cells^18^. Recombinant human CDK1/cyclin B and aurora B were obtained commercially (Cell Signaling). The in vitro kinase assay method was as previously described^18^.

### Analytic gel filtration

Gel filtration was performed on a Superdex-200 10/300 GL column (Amersham Pharmacia Biotech, Orsay, France). HeLa cells were lysed in TNE buffer [10 mmol/l Tris-HCl (pH 7.4), 200 mmol/l NaCl, 1 mmol/l EDTA, 1 mmol/l phenylmethylsulfonyl fluoride (PMSF), and 1 mmol/l dithiothreitol (DTT) containing a protease inhibitor cocktail (Sigma-Aldrich)] and cleared by ultracentrifugation. The sample (500 μl, 20 μg/μl) was loaded onto a Superdex-200 10/300 GL column (10 mm i.d. × 300 mm) equilibrated with TNE buffer. The proteins were fractionated with TNE buffer at a linear flow rate of 0.4 ml/min, and 1-ml fractions were collected.

## Results

### BEX4 is likely incorporated with mitotic kinase in a cell cycle-dependent manner

**Fig. 1:**
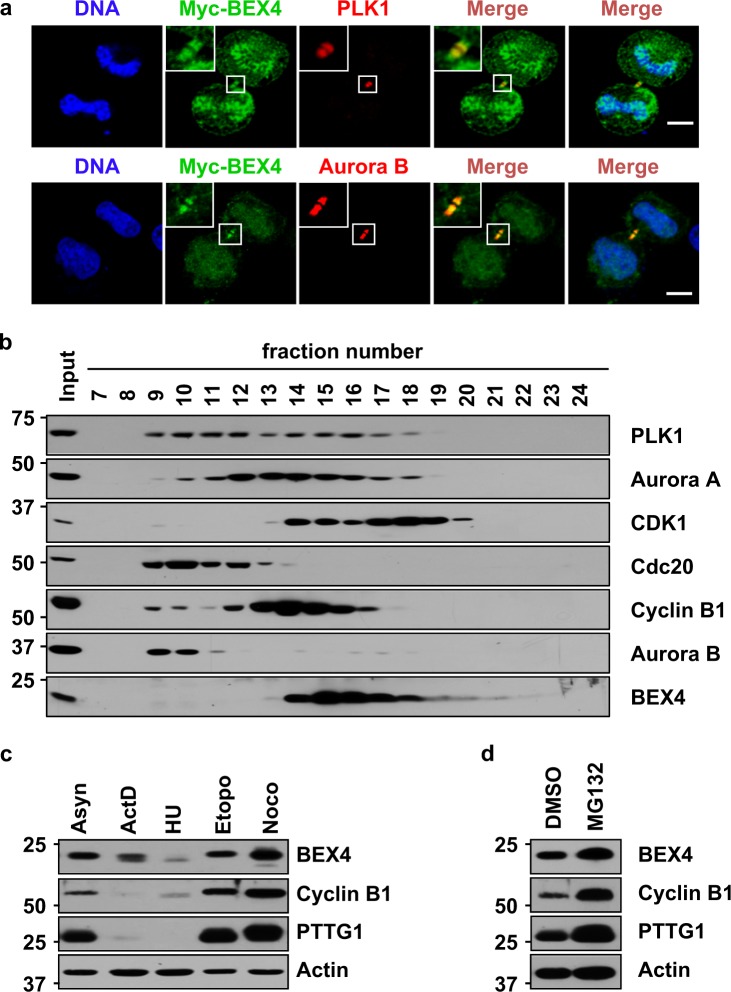
Localization and expression of BEX4 proteins in mitosis. **a** HeLa cells expressing Myc-tagged BEX4 were stained with anti-Myc, anti-PLK1, and anti-aurora B antibodies, as indicated. Scale bars represent 5 μm. The insets show magnified images of cells at midbody. **b** Extracts were prepared from HeLa cells synchronized at mitosis by nocodazole and fractionated by gel filtration using a Superdex-200 column. Fractions (numbered 7–24) were analyzed by immunoblotting using antibodies against PLK1, aurora A, CDK1, Cdc20, cyclin B1, aurora B, and BEX4. Input indicates 4% of the extract. **c** BEX4 expression levels are shown in different phases of the HeLa cell cycle. HeLa cells were synchronized by 5 nM Actinomycin D (ActD, G1-phase), 75 μg/mL hydroxyurea (HU, S-phase), 0.5 μM etoposide (Etopo, G2/M-phase), and 200 ng/ml nocodazole treatment (Noco, M-phase) for 16 h. The lysates were analyzed by immunoblotting using anti-BEX4, anti-cyclin B1, anti-PTTG1, and anti-actin antibodies. **d** HeLa cells were treated with the proteasome inhibitor MG132 (10 μM). After 6 h, the cells were harvested and subjected to immunoblotting with anti-BEX4, anti-cyclin B1, anti-PTTG1, and anti-actin antibodies

Our recent studies revealed that BEX4 is distinctly localized at centrosomes and microtubules from prophase to metaphase and at the midbodies from anaphase to telophase^12^. Mitotic kinases, such as aurora B and PLK1, accumulate in midbodies during the telophase and are essential for spindle assembly/formation, chromosome segregation, and cytokinesis^14^. Our immunofluorescence analysis using HeLa cells transfected with Myc-tagged BEX4 plasmid showed that BEX4 clearly colocalized with endogenous aurora B and PLK1 at midbodies (Fig. 1a).

Gel filtration was then performed to gain further insight into the interaction between PLK1 and BEX4. The endogenous PLK1, aurora A, CDK1, Cdc20, cyclin B1, aurora B, and BEX4 proteins of synchronized HeLa cells by nocodazole treatment for 16 h were size fractionated, and the constituent proteins were identified by immunoblotting (Fig. 1b). We detected both PLK1 and BEX4 in the medium molecular mass range, especially the fractions numbered 14, 15, 16, and 17. However, under the same experimental conditions, aurora B was detected in a higher molecular mass range. These results imply that BEX4 is likely incorporated with the same cellular fraction as PLK1.

To determine the levels of BEX4 during the cell cycle progress, HeLa cells were synchronized by actinomycin D (ActD, G1-phase), hydroxyurea (HU, S-phase), etoposide (Etopo, G2/M-phase), and nocodazole treatment (Noco, M-phase) for 16 h. The lysates were analyzed by immunoblotting using anti-BEX4, anti-cyclin B1, anti-PTTG1, and anti-actin antibodies (Fig. 1c). BEX4 protein was significantly higher in the mitotic phase compared with other phases of the cell cycle. However, the stability of BEX4 was dramatically reduced in S-phase (Fig. 1c). In addition, we tested whether BEX4 was degraded by the proteasome. The HeLa cells were treated with or without MG132 (proteasome inhibitor). As expected, BEX4 protein was increased by MG132 treatment compared with the control (DMSO) treatment (Fig. 1d). Together, these observations suggest that BEX4 is likely incorporated with mitotic kinase, such as CDK1 and PLK1, and its stability is regulated in a cell cycle-dependent manner.

### PLK1 directly interacts with and phosphorylates BEX4 in mitosis

**Fig. 2:**
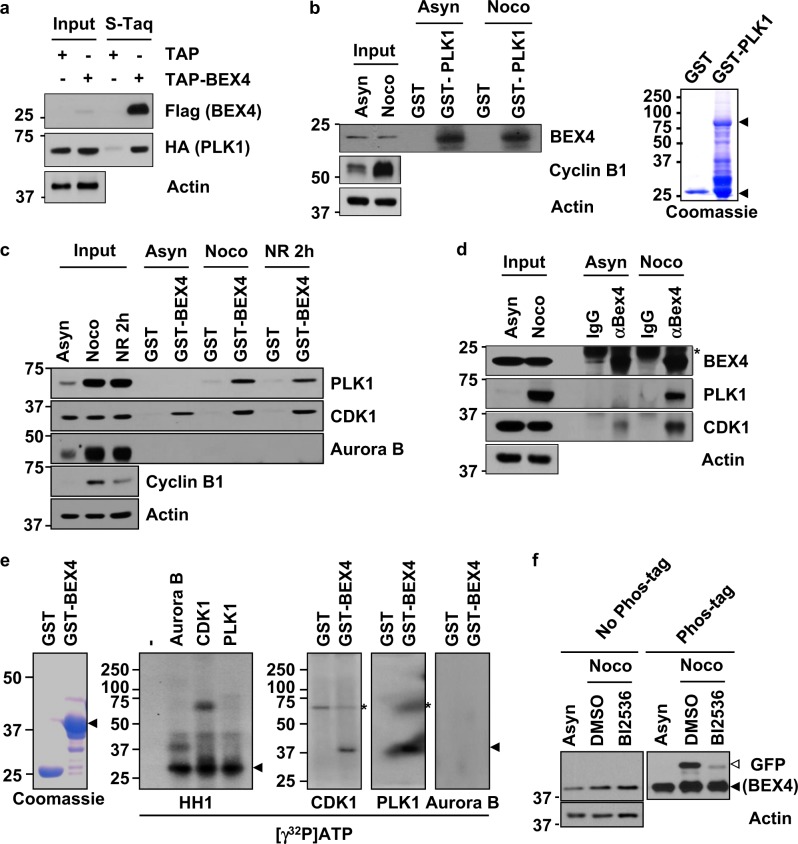
PLK1 directly interacts with and phosphorylates BEX4. **a** Cell lysates from HeLa cells stably expressing TAP and TAP-Pellino-1 were isolated by tandem affinity purification (TAP). Bound proteins were immunoblotted with anti-Flag, anti-PLK1, and anti-actin antibodies. **b** Asynchronized HeLa cells (Asyn) were treated with nocodazole (200 ng/ml) for 16 h (Noco). The HeLa cell extracts of the three different conditions were incubated with beads bound to GST alone or to GST-fused PLK1 proteins. Bound proteins were analyzed by SDS-PAGE and immunoblotted with anti-BEX4, anti-cyclin B1, and anti-actin antibodies. **c** Asynchronized HeLa cells (Asyn) were treated with nocodazole (200 ng/ml) for 16 h (Noco) and transferred to fresh media for 2 h (NR 2 h). The HeLa cell extracts of the three different conditions were incubated with beads bound to GST alone or to GST-fused BEX4 proteins. Bound proteins were analyzed by SDS-PAGE and immunoblotted with anti-PLK1, anti-CDK1, anti-aurora B (Aur B), anti-cyclin B1, and anti-actin antibodies. **d** HeLa cell extracts were obtained from cell cultures not containing (Asyn) or containing (Noco) nocodazole (200 ng/ml). Extracts were immunoprecipitated with normal immunoglobulin IgG or anti-BEX4 antibody, then immunoblotted with anti-BEX4, anti-PLK1, anti-CDK1, and anti-actin. The asterisk represents a nonspecific band. **e** Recombinant CDK1/Cyclin B (CDK1), PLK1, and aurora B (Aur B) proteins were mixed with kinase buffer containing [γ^32^P] ATP. Mixtures were reacted with bead-bound histone H1 (HH1, a positive control), GST, or GST-BEX4, run in SDS-PAGE, and visualized by autoradiography. Coomassie-stained gel is shown for GST and GST-BEX4 proteins. The asterisk represents autophosphorylated CDK1 and PLK1. **f** Detection of phosphorylation of BEX4 by the Phos-tag reagent as described in the Materials and methods. Lysates from GFP-BEX4-expressing stable HeLa cells with or without 60 nM BI2536 (PLK1 inhibitor) in the nocodazole (200 ng/ml)-treated condition for 12 h were separated by SDS-PAGE in the absence (left) or presence (right) of the Phos-tag reagent and immunoblotted with anti-GFP and anti-actin antibodies. The supershifted bands correspond to the phosphorylated BEX4 (open triangle). Unphosphorylated BEX4 is indicated by (solid triangle)

To examine whether PLK1 directly interacts with BEX4, cellular extracts from an asynchronously growing TAP culture and Flag-tagged TAP-BEX4-transfected 293T cells were subjected to pull-down assays, which showed that BEX4 interacted with endogenous PLK1 (Fig. 2a). In addition, GST–PLK1 fusion proteins were incubated with cellular extracts from HeLa cells treated with or without nocodazole (Fig. 2b). The pull-down assays revealed that GST-BEX4 bound to PLK1, particularly in mitotically synchronized cells and to its priming kinase CDK1 (Fig. 2c). However, under the same experimental conditions, BEX4 failed to make a complex with aurora B kinase. We also immunoprecipitated endogenous BEX4 from HeLa cells treated with or without nocodazole, and found that BEX4 formed a complex with PLK1 and CDK1 preferentially in mitotically synchronized cells (Fig. 2d).

To examine whether PLK1 directly phosphorylates BEX4, we incubated GST-BEX4 with purified recombinant PLK1, CDK1/cyclin B, or aurora B in the presence of [γ^32^P]-ATP (Fig. 2e). Although PLK1 and its priming kinase CDK1/cyclin B efficiently phosphorylated BEX4, aurora B kinase failed to do so. In addition, we found that the inhibition of PLK1 activity by BI2536 (selective PLK1 inhibitor) treatment dramatically reduced the phosphorylation of BEX4 in nocodazole (Noco; 200 ng/ml)-treated HeLa cells stably expressing GFP-BEX4 (Fig. 2f). Together, these observations suggest that PLK1 interacts with and phosphorylates BEX4.

### PLK1-mediated phosphorylation contributes to the stability and localization of BEX4

**Fig. 3:**
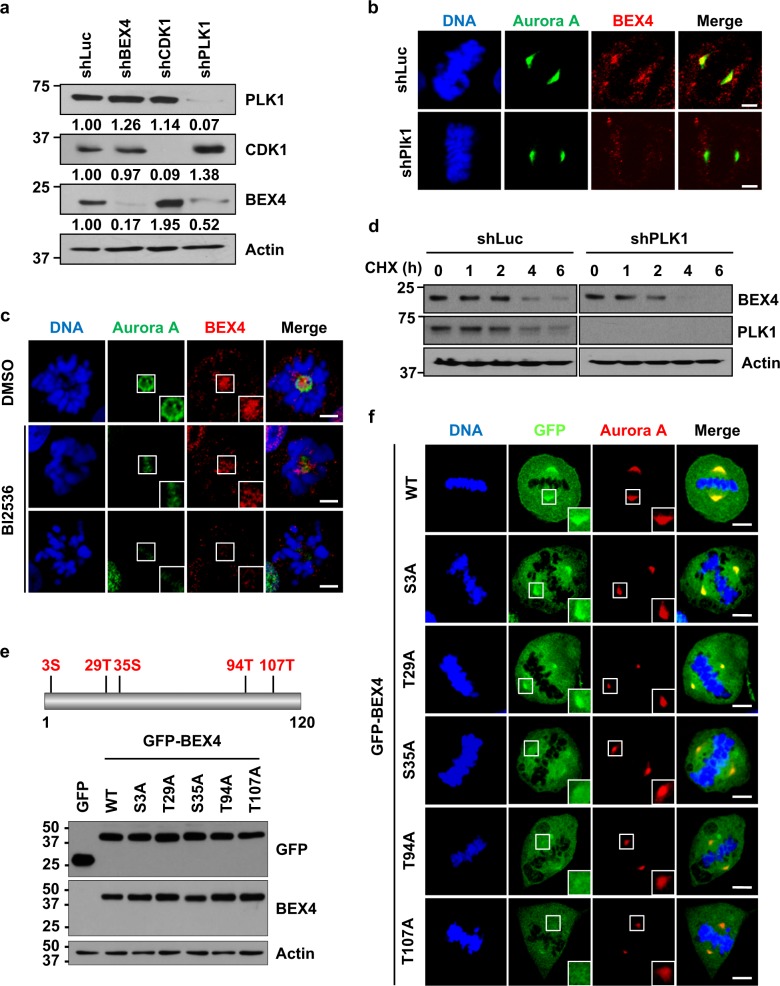
PLK1 phosphorylates BEX4 and regulates its subcellular localization during mitosis. **a** HeLa cells were transfected with shLuc, shBEX4, shCDK1, or shPLK1 and cultured in the presence of nocodazole (200 ng/ml) for 16 h. The cells were collected by shake-off, then lysed and immunoblotted with anti-PLK1, anti-CDK1, anti-BEX4, and anti-β-actin. Expression levels relative to respective controls are shown below the blots. **b** HeLa cells were transfected with control shLuc or PLK1-targeting shRNA, cultured on coverslips for 24 h and stained with anti-aurora A (Aur A), anti-BEX4, and DAPI. The metaphase cells were then analyzed. Scale bars represent 5 μm. **c** HeLa cells were cultured on coverslips for 24 h, then treated with BI2536 (300 nmol/l; a selective PLK1 inhibitor) or DMSO as the control. At 2 h post-treatment, the cells were fixed with 4% paraformaldehyde and stained with anti-BEX4 and anti-aurora A (Aur A) antibodies. DNA was visualized by DAPI staining. The insets show magnified images of centrosomes. Scale bars represent 5 μm. **d** HeLa cells were transfected with shLuc or shPLK1. At 36 h after transfection, the cells were treated with cycloheximide (CHX) for the indicated times and then subjected to immunoblotting with anti-BEX4, anti-PLK1, and anti-actin antibodies. **e**, **f** All serine (S) and threonine (T) residues in human BEX4 protein. HeLa cells were transfected with GFP-fused BEX4 mutant constructs with serine and/or threonine substituted with alanine (S3A, T29A, S35A, T94A, or T107A), or a BEX4 wild-type construct. The expression of each BEX4 expression construct was analyzed by immunoblotting using anti-GFP, anti-BEX4, and anti-actin antibodies (**e**). Cells were fixed with 4% paraformaldehyde and stained with anti-GFP and anti-aurora A (Aur A) antibodies. DNA was visualized by DAPI staining (**f**). The insets show magnified images of the centrosomes. Scale bars represent 5 μm

To analyze the function of the physical interaction between BEX4 and PLK1, we transfected HeLa cells with shPLK1 or shCDK1 and then cultured cells in the presence of nocodazole (Fig. 3a). Interestingly, depletion of PLK1 clearly reduced BEX4 protein, but CDK1 depletion did not. Further immunofluorescence analysis revealed that PLK1 depletion sharply reduced BEX4 protein, which was localized at the centrosomes (Fig. 3b). In addition, the inhibition of PLK1 activity by BI2536 treatment sharply reduced BEX4 protein, which was localized at the centrosomes in the HeLa cells or the GFP-BEX4 stable cell lines (Fig. 3c; Figure S1).

We then compared the stability of BEX4 protein in asynchronized PLK1-depleted HeLa cells by CHX treatment, a protein biosynthesis inhibitor (Fig. 3d). As expected, BEX4 rapidly declined and was almost undetectable 4 h post-treatment in the PLK1-depleted (shPLK1) cells, whereas BEX4 was slightly reduced but still detectable 6 h post-treatment in the control (shLuc) cells (Fig. 3d). These results indicate that the downregulation of PLK1 promotes the degradation of BEX4 protein.

To determine whether PLK1-mediated phosphorylation affects the centrosome localization of BEX4, we constructed plasmids encoding GFP-tagged BEX4 mutants, in which a single serine or threonine residue of BEX4 was changed to alanine (S3A, T29A, S35A, T94A, and T107A) (Fig. 3e), and then transfected them into HeLa cells (Fig. 3e, f). Interestingly, the GFP-BEX4 S3A, T29A, S35A, and T94A mutants were localized to centrosomes or spindle poles similar to GFP-BEX4 WT, but the GFP-BEX4 T107A mutant exhibited a diffuse distribution around the centrosomes (Fig. 3f). Furthermore, when prometaphase cells were treated with BI2536, the BEX4 at the centrosomes was significantly reduced (Figure S1), indicating that the PLK1-mediated phosphorylation of BEX4 appears to be essential for its localization at centrosomes. Taken together, these results indicate that the PLK1-mediated phosphorylation is involved in the stability and centrosome localization of BEX4.

### BEX4 expression augments the generation of aneuploid cells by reducing the apoptotic cell death

**Fig. 4:**
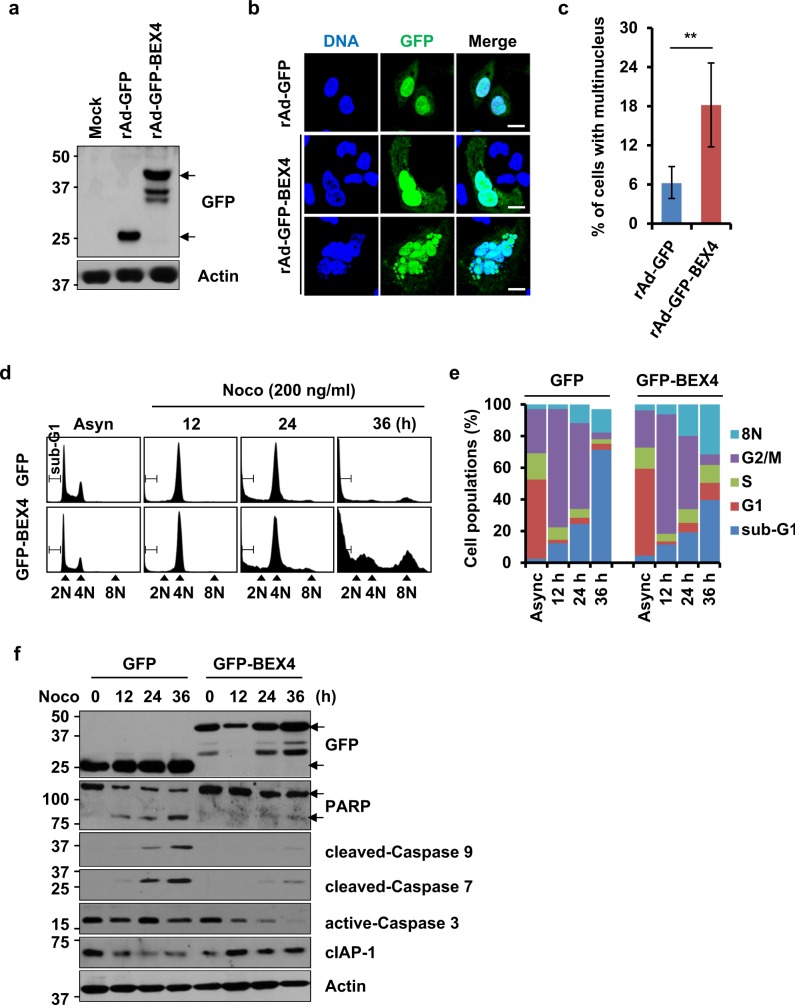
BEX4 expression leads to aneuploidy adaptation in response to mitotic spindle damage. **a** A549 cells were infected with adenovirus (rAd) expressing a control GFP or GFP-BEX4, and the levels of GFP and GFP-BEX4 were analyzed by immunoblotting. **b** A549 cells expressing GFP or GFP-BEX4 were fixed with 4% paraformaldehyde, and stained with DAPI to visualize DNA. Scale bars represent 10 μm. **c** The percentage of multinucleated cells was calculated using at least 100 cells per experiment. **P < 0.01. **d** Doxycycline-treated GFP- or GFP-BEX4-inducible cells were treated with nocodazole (200 ng/ml). At the times indicated, the cells were harvested and stained with propidium iodide. DNA contents were analyzed by flow cytometry. **e** Histograms summarizing the distribution of different DNA contents in cells analyzed in (**a**). The results are the mean ± SEMs of three independent experiments. **f** Immunoblots with anti-GFP, anti-poly(ADP-ribose) polymerase-1 (PARP), anti-cleaved-caspase 9, anti-cleaved-caspase 7, anti-cleaved-caspase 3, anti-cIAP-1, and anti-β-actin antibodies in the cells analyzed in (**d**)

PLK1 is essential for spindle assembly/formation and chromosome segregation^14^, and overexpression of constitutively active PLK1 with T210D mutation causes polyploidy^22^. As the stability of BEX4 was regulated by PLK1 activity (Fig. 3), we examined whether ectopic expression of BEX4 induces polyploidy in cancer cells. We generated recombinant adenovirus (rAd) expressing GFP or GFP-BEX4 and infected them into A549 lung cancer cell (Fig. 4a). Immunofluorescence analysis showed that BEX4 overexpression significantly increased the multinucleated cells compared with the control cells (Fig. 4b, c), indicating that aberrant expression of BEX4 promotes the aneuploidy formation in lung cancer cells.

To determine whether BEX4 expression affects the mitotic checkpoint and chromosome segregation, HeLa cells overexpressing BEX4 were cultured in the absence (Asyn) or presence of nocodazole. Most of the control GFP-expressing cells that had been arrested at mitosis by nocodazole eventually died (Fig. 4d, sub-G1 population), whereas the remaining cells exited cell cycle arrest in a process called “mitotic adaptation” or “mitotic slippage” and became aneuploid (Fig. 4d, e, 8N population)^10,23,24^. However, the extended incubation of BEX4-overexpressing cells in the presence of nocodazole led to the marked accumulation of aneuploid cells and relatively few apoptotic cells. These observations suggest that chromosomal aneuploidy in response to spindle damage seems to be directly regulated by BEX4 protein level.

After finding that BEX4 expression allowed abnormal mitotic cells to adapt to mitotic spindle damage, we investigated whether the BEX4-induced aneuploidy adaptation directly couples with anti-apoptotic cell death. Interestingly, the expression of GFP-BEX4 significantly decreased cleaved-PARP, active-caspase 9, active-caspase 7, and active-caspase 3 but augmented cIAP-1 in response to nocodazole treatment (Fig. 4f). These results suggest that BEX4 functions as an anti-apoptotic molecule during the acquisition of aneuploidy.

### PLK1 directly phosphorylates BEX4 at T107 and contributes to BEX4-induced aneuploidy

**Fig. 5:**
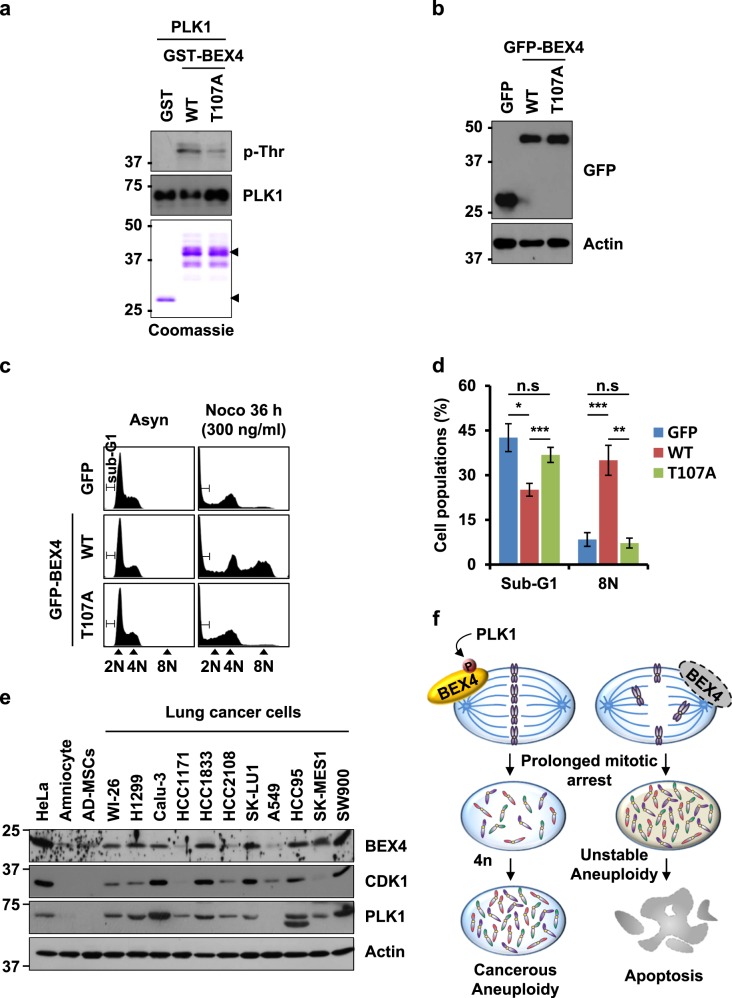
PLK1-associated phosphorylation at T107 regulates the oncogenic function of BEX4. **a** Recombinant PLK1 protein was incubated with bead-bound GST, GST-BEX4 WT, or GST-BEX4 T107A mutant in the presence of ATP. Mixtures were immunoblotted with the indicated antibodies. Coomassie-stained gel is shown for GST, GST-BEX4 WT, and GST-BEX4 T107A mutant proteins. Arrowheads indicate GST, GST-BEX4 WT, and GST-BEX4 T107A. **b** HeLa cells were transfected with GFP-, GFP-BEX4 WT-, or GFP-BEX4 T107A-expressing plasmids. Each sample was immunoblotted with the indicated antibodies. **c** HeLa cells expressing GFP, GFP-BEX4 WT, or GFP-BEX4 T107A were treated with nocodazole (300 ng/ml). After 36 h, the cells were harvested and stained with propidium iodide. DNA contents were analyzed by flow cytometry. **d** Histograms summarizing the distribution of different DNA contents in the cells analyzed in (**c**). The results are the mean ± SEMs of three independent experiments. *P<0.05, **P<0.01, ***P<0.001. **e** HeLa cells, two non-tumor cell lines [amniocyte and adipose-derived mesenchymal stem cells (AD-MSCs)] and 11 lung cancer cell lines (WI-26, H1299, Calu-3, HCC1171, HCC1833, HCC2108, SK-LU-1, A549, HCC95, SK-MES-1, and SW900) were lysed and immunoblotted with anti-BEX4, anti-CDK1, anti-PLK1, and anti-actin antibodies. **f** A model of the functions of BEX4 during mitosis. Refer to the Discussion section for details

Phosphorylation of BEX4 on T107 was important for proper BEX4 localization, and the inhibition of PLK1 altered the centrosomal localization of BEX4 (Fig. 3). To determine whether PLK1 directly phosphorylates BEX4 at T107, the recombinant GST-BEX4 WT and GST-BEX4 T107A mutant were expressed in *E. coli* and purified. We incubated GST-BEX4 and its mutant with recombinant PLK1, and threonine phosphorylation was detected by immunoblotting using anti-phospho threonine (p-Thr). Although PLK1 efficiently phosphorylated GST-BEX4 WT, the threonine phosphorylation of GST-BEX4 T107A was significantly decreased, suggesting that T107 in BEX4 is phosphorylated by PLK1 (Fig. 5a).

Given the diffused localization of GFP-BEX4 T107A around centrosomes (Fig. 3f), we assessed if the T107A mutant failed to induce mitotic adaptation. We transfected HeLa cells with GFP-BEX4 WT or T107A mutant, then cultured cells in the presence of nocodazole (Figs. 5b, c). By 36 h after nocodazole treatment, GFP-BEX4 WT dramatically increased the population of aneuploid cells (8N population). However, overexpression of GFP or GFP-BEX4 T107A resulted in a significantly increased sub-G1 population and relatively few aneuploid cells (Fig. 5c, d), indicating T107A was far weaker than WT in its anti-apoptotic functions. These results suggest that PLK1 had considerable influence on the induction of chromosomal aneuploidy in response to spindle damage by phosphorylating and stabilizing BEX4.

Growing evidence indicates that PLK1 is overexpressed in a wide range of tumors and is often associated with poor prognosis^15^. Therefore, we examined the relationships between BEX4 expression and PLK1 and CDK1 expression in human lung cancer cells. Immunoblotting analysis revealed that PLK1 was overexpressed in 10 of the 11 lung cancer cells, and unexpectedly, 9 of the lung cancer cell lines showed a significant positive correlation between the expression of BEX4 and the expression of PLK1 or CDK1 (Fig. 4d). Together, these data indicate that PLK1 functionally cross-talks with BEX4 and may change the biochemical properties of BEX4 with respect to the oncogenic potential of PLK1 (Fig. 4e).

## Discussion

This study, which was undertaken to elucidate the molecular function of BEX4 in tumorigenesis, shows that BEX4 interacts with PLK1, and this BEX4–PLK1 interaction is a novel oncogenic signal that enables the acquisition of aneuploidy adaptation. BEX4 protein predominantly localizes at many mitotic features, such as centrosomes, contractile rings, and midbodies, and interestingly plays an important role in the stabilization of microtubules, affecting their elongation rates. Furthermore, BEX4 expression appears to be critical for determining whether cells undergo apoptosis or adapt to aneuploidy induced by spindle damage or a mitotic checkpoint defect (Fig. 5f).

Centrosomes in mitosis orchestrate the assembly and functioning of mitotic spindles and ensure the equal partitioning of the replicated genome into daughter cells. Thus, centrosome dysfunction is directly linked to aneuploidy, which is particularly prevalent in tumor cells^25^. However, it remains unanswered whether centrosome defects alone can cause cancer. Nevertheless, it was recently recognized that the manipulation of molecular networks controlling the centrosome function represent important targets for specific therapeutic intervention in cancer, because normal cells usually lack centrosome alterations^26^. The present study shows that the aberrant expression of BEX4 deregulated microtubule elongation from centrosomes and microtubule stability. BEX4 is also recruited to contractile rings and midbodies during chromosome separation. Furthermore, the stability of BEX4 was directly regulated by PLK1-mediated phosphorylation, and it was highly correlated with the status of PLK1 expression in human lung cancer cell lines compared with non-tumor normal cell lines, including amniocyte and adipose-derived mesenchymal stem cells (AD-MSCs) (Fig. 5e). Interestingly, PLK1 is overexpressed in a wide range of tumors and is often associated with a poor prognosis^15^. Therefore, it seems that PLK1-mediated phosphorylation causes the oncogenic modification of BEX4 protein and disrupts microtubule elongation and stabilization during mitosis.

The present study also shows that BEX4 expression confers a proliferative advantage on abnormal mitotic cells and protects them against spindle damage-induced cell death. Earlier studies suggested that the inappropriate signaling to stabilize centrosomes and organize their microtubules leads to aberrant cell cycle regulation, overriding of the mitotic checkpoint, and chromosome aneuploidy^5^. These suggestions indicate that the finely tuned regulation of microtubule dynamics may protect cells from excess microtubule damage that might occur during sustained mitotic arrest and compromise cell survival if cells fail to return to mitotic cell cycle progression. Interestingly, BEX4 expression reduced the population of nocodazole-induced apoptotic cells but increased the population of the aneuploid cells via oncogenic transformation. Thus, BEX4 expression needs to be stringently regulated by a yet-unidentified mechanism. However, the stabilization of BEX4 by the constitutive expression of PLK1 resulted in severe mitotic defects, which are associated with cancer.

The distinct subcellular localizations of PLK1 at various subcellular structures during mitotic progression is unusual, such as its association with centrosomes in prophase, enrichment at kinetochores in prometaphase and metaphase, recruitment to central spindles in anaphase, and accumulation in midbodies during telophase^14^. In the present study, it was confirmed that PLK1 interacts with and phosphorylates BEX4 and that BEX4 and PLK1 colocalize at midbodies (Fig. 1a). This finding and the observation that BEX4 expression is highly correlated with the expressional level of PLK1 in cancer cells indicate that the interaction and colocalization of BEX4 and PLK1 could be a key oncogenic signal. In summary, this study reveals that BEX4 and PLK1 interaction acts as a novel oncogenic signal, which leads to the acquisition of aneuploidy transformation, and thus, the regulation of BEX4 expression could be a potential therapeutic strategy.

## Electronic supplementary material

Supplementary Figure and Figure Legend
