# Supplementary material for: Oncogenic potential of BEX4 is conferred by Polo-like kinase 1-mediated phosphorylation
Source: Exp Mol Med. 2018 Oct 22;50(10):138. doi: 10.1038/s12276-018-0168-0 (PMC6203768; doi:10.1038/s12276-018-0168-0)
Supplement: Supplementary file 1 — Supplementary Figure and Figure Legend [file 12276_2018_168_MOESM1_ESM.pptx]

## Slide 1
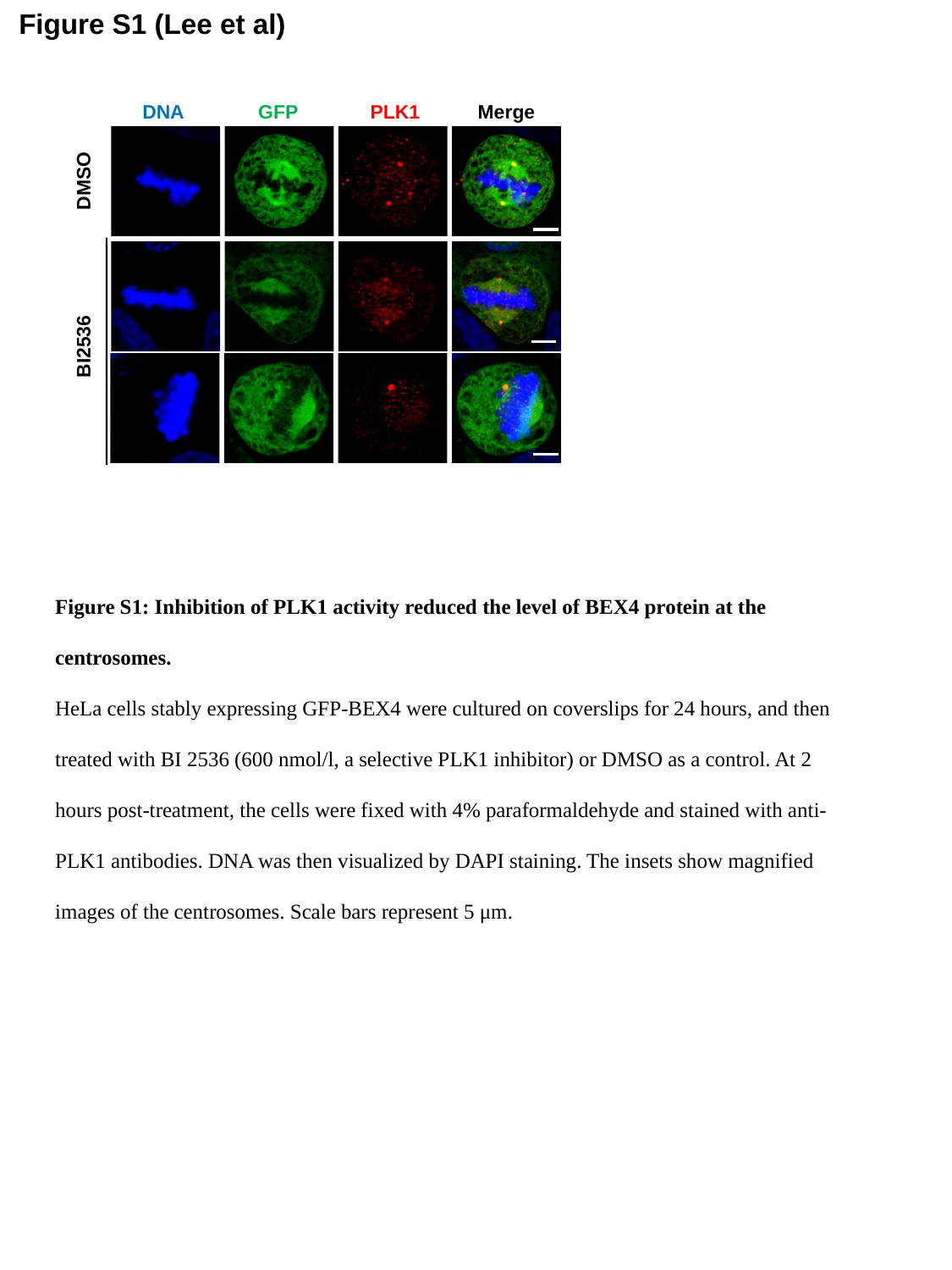

Figure S1 (Lee et al)
PLK1
DNA
GFP
Merge
DMSO
BI2536
Figure S1: Inhibition of PLK1 activity reduced the level of BEX4 protein at the centrosomes.
HeLa cells stably expressing GFP-BEX4 were cultured on coverslips for 24 hours, and then treated with BI 2536 (600 nmol/l, a selective PLK1 inhibitor) or DMSO as a control. At 2 hours post-treatment, the cells were fixed with 4% paraformaldehyde and stained with anti-PLK1 antibodies. DNA was then visualized by DAPI staining. The insets show magnified images of the centrosomes. Scale bars represent 5 μm.
